# Effect of Adding Konjac Glucomannan on the Physicochemical Properties of Indica Rice Flour and the Quality of Its Product of Instant Dry Rice Noodles

**DOI:** 10.3390/foods13233749

**Published:** 2024-11-22

**Authors:** Chunmiao Lu, Ying Yang, Xin Zhao, Zhiyu Liu, Xiaoyan Liao, Yingying Zhang, Dailin Wu, Jing Li, Jiangtao Li

**Affiliations:** National Engineering Research Center of Rice and Byproduct Deep Processing, College of Food Science and Engineering, Central South University of Forestry and Technology, Changsha 410004, China; luchunmiao99@163.com (C.L.); zxi0416@163.com (X.Z.); uyihziu@163.com (Z.L.); yizhiyang1027@163.com (X.L.); zy_zy1214@163.com (Y.Z.); 18144404523@163.com (D.W.); 17872399675@163.com (J.L.); ljthyd@163.com (J.L.)

**Keywords:** instant dry rice noodles, Indica rice flour, konjac glucomannan, physicochemical properties, quality

## Abstract

Instant dry rice noodles have a broad market prospect due to their advantages of long shelf life, convenient transportation, and convenient eating, but there are still quality problems such as long rehydration times and poor eating quality. In order to improve the quality of instant dry rice noodles, the effects of konjac glucomannan (KGM) on the gelatinization characteristics, pasting properties, and rheological properties of Indica rice flour and the structure, food quality, and starch digestibility of instant dry rice noodles made of Indica rice flour were studied. The results showed that the starch gelatinization conclusion temperature and endothermic enthalpy of Indica rice flour were reduced by adding ≤ 3% KGM, the peak viscosity, valley viscosity, final viscosity, and setback value of Indica rice flour in the pasting process decreased with the increase in the KGM addition amount, and the pseudoplasticity, viscosity, and elasticity of Indica rice flour paste were reduced by adding > 1% KGM. When the KGM addition amount was 2%, the endothermic enthalpy, final viscosity, and setback value of Indica rice flour were 2.74 J/g, 2379.5 cp, and 961.5 cp, respectively. The instant dry rice noodles made of Indica rice flour had a looser microstructure after adding KGM, and its short-range ordered structure and double helix content were reduced by adding 1~3% KGM. When the KGM addition amount was 2%, the rehydration time of instant dry rice noodles was 290 s, which was shortened by 14.7%, while the texture and sensory quality remained unchanged, and the SDS content was reduced by 16.4% while the RS content was increased by 28.8%. Therefore, the physicochemical properties of Indica rice flour and the quality of its instant dry rice noodles can be improved by adding an appropriate amount of KGM. This study can promote the application of KGM in improving the quality of rice products.

## 1. Introduction

Rice noodles are a traditional staple food made from rice that is often processed by milling, pasting, and extrusion. Indica rice with high amylose content has been proved to be more suitable for processing into rice noodles [1]. According to the difference in water content, rice noodles can be divided into instant fresh wet rice noodles, semi-dry rice noodles, and dry rice noodles. Ordinary dry rice noodles generally need to be soaked and cooked for a long time to eat, while instant dry rice noodles can be eaten after being brewed with hot water for several minutes. Obviously, in addition to long shelf life and convenient transportation, instant dry rice noodles also have the advantages of convenient eating, so they have a broad prospect in the global rice noodle market of up to 1 billion USD/year. However, there are few instant dry rice noodles on the market at present, and the core technical problem to be solved in developing the products is to shorten the rehydration time of the dry rice noodles. Now, the reported method for improving rehydration characteristics of dry rice noodles is to pretreat rice flour. For some rice flour, after mild steaming and drying for 12 h [2], being modified by a 1,4-α-glucan branching enzyme at 50 °C for 12 h [3], or soaking for 4–6 h followed by draining, wet-milling, drying at 45 °C for 6–8 h, crushing, and adding 20% of erythritol (*w*/*w*) [4], the rehydration time of dry rice noodles processed from it can be shortened by 26% to 44%. However, the pretreatment of rice flour will greatly increase the production costs of dry rice noodles, and adding a large amount of erythritol is likely to reduce the natural flavor of rice noodles.

Konjac glucomannan (KGM) is a water-soluble, non-ionic polysaccharide extracted from konjac tubers [5]. As a dietary fiber and non-caloric food ingredient, KGM plays an important role in reducing weight, cholesterol, and blood pressure and altering metabolism of the gut microbiota [6]. In addition, KGM is widely used as a food additive in food processing not only because of its good thickening and gelling properties but also because of its extremely strong water absorption [7]. Previous studies have shown that adding KGM can increase the water retention of wheat gluten [8], increase the water absorption of wheat dough [9], reduce the hardness of steamed bread [10], and reduce the cooking loss of noodles [11]. It can be seen that KGM may improve the rehydration characteristics of dry rice noodles due to its strong water absorption, but there is no relevant research report. Regarding the research on the effect of KGM on rice products, it has been reported that adding appropriate amounts of KGM can improve the food quality of rice porridge [12] and mochi [13]. In order to confirm that KGM can improve the rehydration characteristics of dry rice noodles and to provide a theoretical basis for the application of KGM in rice product processing, this paper studies the effect of adding KGM on the physicochemical properties of Indica rice flour and the quality of its product of instant dry rice noodles. Due to the KGM addition amount in starch and starchy foods being generally less than 4% [12,14], this study set the addition level of KGM at 1–4%.

## 2. Materials and Methods

### 2.1. Materials

Zhenzhu 3 Indica rice was produced in Nanling, Wuhu, Anhui, China, processed to grade 2 accuracy, sealed, and stored at room temperature after processing. Its total starch, amylose, protein, and fat contents were 79.69%, 16.37%, 8.59%, and 0.57%, respectively. KGM with a purity of 90% was purchased from Henan Wanbang Industrial Co., Ltd. (Zhengzhou, China). Calcium chloride, acetic acid, sodium acetate, hydrochloric acid, and other analytically pure chemical reagents were purchased from Shanghai National Pharmaceutical Group Chemical Reagent Co., Ltd. (Shanghai, China). α-Amylase (A3176, 10 U/mg) and pepsin (P7125, 400 U/mg) were purchased from Sigma Reagent Co., Ltd. (St. Louis, MO, USA). α-1,4-Glucoside amylase (S10018, 100,000 U/mg) was purchased from Shanghai Yuanye Biotechnology Co., Ltd. (Shanghai, China) GOPOD reagent was purchased from Megazyme Co., Ltd. (Wicklow, Ireland).

### 2.2. Preparation of Indica Rice Flour with Different Amounts of KGM

Indica rice was milled and passed through a 100-mesh sieve to obtain Indica rice flour. Moreover, 1%, 2%, 3%, and 4% of KGM (*w*/*w*) were added into Indica rice flour and mixed evenly to obtain Indica rice flour samples with different amounts of KGM. All the samples were sealed and stored at room temperature.

### 2.3. Determination of Thermal Properties of Flour Samples

The thermal properties of the flour samples were determined using a differential scanning calorimeter (DSC Q2000, TA Instruments Co., Ltd., New Castle, DE, USA). According to the method described in the reference [15] with minor modifications, 3.0 mg of the flour samples and 6.0 mL of water were sealed in an aluminum crucible and equilibrated at 4 °C for 24 h, and the testing conditions were heating from 30 °C to 120 °C at a rate of 10 °C/min.

### 2.4. Determination of Pasting Properties of Flour Samples

The pasting properties of the flour samples were determined using a rapid viscosity analyzer (RVA, SUPER-4, Poton Co., Ltd., Stockholm, Sweden) and the standard 1 setting procedure of AACC 76-21 [16].

### 2.5. Determination of Rheological Properties of Flour Paste Samples

Rheological properties of the flour paste samples produced by RVA analysis in 2.4 were determined using a rheometer (DHR-2 dynamic rheometer, Waters Group Co., Ltd., Milford, MA, USA) and a 40 mm and 2° cone plate.

The flow properties of the flour paste samples were tested using a steady shear procedure with shear rates ranging from 0.1 s^−1^ to 1000 s^−1^ at 25 °C and described using the rheological parameters n and K of the Herschel–Bulkley model as shown by the following formula [17]:

(1) $$\sigma =\sigma_{0}+K{\overset{\cdot }{\gamma }}^{n}$$

where σ, σ_0_, K, $\overset{\cdot }{\gamma }$, and n are the shear stress (Pa), yield stress (Pa), consistency coefficient (Pa·s), shear rate (s^−1^), and flow behavior index (dimensionless), respectively.

The viscoelasticity of the flour paste samples was tested using a frequency sweep procedure of 0.1~100 rad/s at 25 °C in the linear strain range of the samples.

### 2.6. Preparation of Instant Dry Rice Noodles

Instant dry rice noodles were prepared by mixing each flour sample with water (60%, *w*/*w*) evenly, steaming and extruding the mixture into rice noodles using a rice noodle machine (SZ-30, Guangzhou Xuzhong Food Machinery Co., Ltd., Guangzhou, China) with a mold aperture diameter of 2 mm, aging at 25 °C for 2 h, and then drying at 60 °C for 90 min to make the moisture content of the samples below 13%.

### 2.7. Scanning Electron Microscopy (SEM) Observation of Instant Dry Rice Noodles

The instant dry rice noodles prepared in 2.6 were milled through a 100-mesh sieve to obtain powder samples. The powder samples were fixed on a metal column by a conductive adhesive, sprayed with gold, and then observed using a scanning electron microscope (TESCAN MIR4, Tescan Co., Ltd., Brno, Czech Republic).

### 2.8. X-Ray Diffraction Analysis of Instant Dry Rice Noodles

The crystalline structures of instant dry rice noodles were analyzed using an X-ray diffractometer (Rigaku Smart Lab SE, Rigaku Co., Ltd., Tokyo Met., Japan). The powder samples prepared in 2.7 were scanned from 4° to 60° 2θ at a rate of 10°/min at 40 kV and 50 mA.

### 2.9. Fourier Transform Infrared (FTIR) Spectroscopy Analysis of Instant Dry Rice Noodles

FTIR spectrograms of instant dry rice noodles were obtained by using a FTIR spectrometer (IRTracer-100, Shimadzu Enterprise Management Co., Ltd., Kyoto, Japan) to scan the powder samples prepared in 2.7. In detail, 2.0 mg of the powder samples were fully milled with 200.0 mg of KBr and compressed into tablets. The tablet samples were scanned from 4000 to 500 cm^−1^ at a resolution of 4 cm^−1^.

### 2.10. Measurements of Rehydration Time and Rehydration Loss Rate of Instant Dry Rice Noodles

The rehydration time of instant dry rice noodles was determined according to the method described by Zhu et al. [18] with minor modifications. Samples were brewed with 20 times the weight of hot water and taken out for observation every 10 s after 4 min. The first time to observe the disappearance of a white hard core of the noodles was the rehydration time of instant dry rice noodles.

The rehydration loss rate of instant dry rice noodles was determined by brewing the dry rice noodles with 20 times the weight of hot water to their rehydration time, drying the liquid at 105 °C to a constant weight, and calculating the weight ratio of dry matter in the liquid to the dry rice noodles according to the weight of dry basis.

### 2.11. Texture Analysis of Instant Dry Rice Noodles

The instant dry rice noodles were brewed with 20 times the weight of hot water to their rehydration time to obtain ready-to-eat rice noodles. The texture of the ready-to-eat noodles was tested using a texture analyzer (TA. XT, Stable Micro Systems Co., Ltd., London, UK) with a P/36 R probe, and the testing conditions were 1 mm/s of pre-test, mid-test, and post-test speeds, 75% of compression ratio, 5.0 g of trigger force, and 5 s of compression interval.

### 2.12. Sensory Evaluation of Instant Dry Rice Noodles

Ten volunteers who are physically healthy, feel normal, and have undergone training were invited to taste the ready-to-eat noodles and rate them according to the criteria in Table 1. All volunteers were clearly informed of the experiment content, voluntarily participated in the experiment, and agreed to have the collected data statistically analyzed and published. During the experiment, the participants who tasted the samples did not pose any ethical risks, and their privacy and rights were protected.

### 2.13. Determination of Starch Digestibility of Instant Dry Rice Noodles

The starch digestibility of instant dry rice noodles was determined according to the in vitro digestion method described by Englyst et al. [19]. The ready-to-eat noodles in pH 6.8 acetate buffer solution (0.2 mol/L) were shaken at a reciprocating speed of 150 times per minute at 37 °C using a water bath shaker. Moreover, 1 mL of α-amylase solution (75 U/mL) was added to react for 2 min, and then 1 mol/L of hydrochloric acid solution was quickly added to adjust the pH to 2.0, followed by adding 1 mL of pepsin reaction solution (2000 U/mL) to react for 120 min. Next, 0.2 mol/L of sodium acetate solution was added to adjust the pH to 5.2, and 3 mL of enzyme solution containing α-amylase (35 U/mL) and α-1,4-glucoside amylase (35 U/mL) was added to react for 5 h. When the amylase hydrolysis reaction reached 0, 20, 60, 90, and 120 min, 0.1 mL of the reaction solution was immediately taken out to inactivate the enzyme by keeping in boiling water for 15 min, and then tested glucose content using a GOPOD kit. The digestibility and contents of rapidly digestible starch (RDS), slowly digestible starch (SDS), and resistant starch (RS) of the starch in instant dry rice noodles were calculated according to the following formulas:

(2) $$\mathrm{Digestibility}=\frac{G120\times 0.9}{\mathrm{TS}}\times 100\%$$

(3) $$\mathrm{RSD}=(G20-G0)\times \frac{0.9}{\mathrm{TS}}\times 100\%$$

(4) $$\mathrm{SDS}=\left(G120-G20\right)\times \frac{0.9}{\mathrm{TS}}\times 100\%$$

(5) $$\mathrm{RS}=\frac{\left[\mathrm{TS}-\left(\mathrm{RDS}+\mathrm{SDS}\right)\right]}{\mathrm{TS}}\times 100\%$$

In the formulas, G0, G20, and G120 represent the glucose content of the reaction solution hydrolyzed by amylase for 0, 20, and 120 min, respectively. TS is the total starch content of the samples, equivalent to the glucose content of the reaction solution hydrolyzed by amylase for 5 h.

### 2.14. Data Analysis

Experimental data were processed using Excel software (Redmond, WA, USA) and presented as the average of duplicate or triplicate determinations. One-way analysis of variance and significance tests using Duncan’s multiple comparison method were processed using SPSS 19.0 software (Chicago, IL, USA), and *p* < 0.05 was considered to have a significant difference. Figures were drawn using Origin 2020 software (Northampton, MA, USA). Specifically, FTIR spectrograms were analyzed using OMNIC 8.0 software (Madison, WI, USA).

## 3. Results and Discussion

### 3.1. Effect of Adding KGM on the Starch Gelatinization Characteristics of Indica Rice Flour

Differential scanning calorimetry (DSC), as a commonly used method for measuring starch gelatinization, was used to analyze the effect of adding KGM to starch gelatinization characteristics of Indica rice flour. For all the samples, during the process of heating from 30 °C to 120 °C, only one endothermic peak appeared at around 75 °C. Since the gelatinization temperature of rice starch measured by DSC is generally in the range of 60–85 °C [20], the existing endothermic peak was produced by the gelatinization of starch in Indica rice flour, and the gelatinization parameters are shown in Table 2. The addition of KGM had no significant effect on the gelatinization onset temperature (T_o_) and peak temperature (T_p_) of the starch, but when the addition amount was less than or equal to 3%, it reduced the gelatinization conclusion temperature (T_c_) and endothermic enthalpy (ΔH) of the starch, and when the addition amount was 4%, it did not change T_c_ but increased ΔH by 36.9%. As the stability of the starch amorphous region, crystalline region, and subcrystalline region can be reflected by T_o_, T_p_, and T_c_ of the starch, respectively, and the energy required to destroy the double helix structure of starch in the gelatinization process can be reflected by the corresponding ΔH [21], the DSC results indicate that the addition of KGM mainly affects the thermal degradation of starch in the subcrystalline region and the double helix structure of starch.

Previous studies have shown that the interaction between KGM and starch molecules forms an intermolecular hydrogen bond [22], and the hydrophilicity of KGM limits the water availability for starch gelatinization [23]. It seems that in the gelatinization process of starch in Indica rice flour with the ratio of flour to water by weight of 1:2, the dominant role of KGM in influencing T_c_ and ΔH will change from hydrogen bonding with starch molecules to hydrophilicity limiting water availability with the increase in KGM addition, and the addition amount inducing the change is 3%. Therefore, when the addition amount is less than or equal to 3%, the hydrogen bonding between KGM and starch molecules is strong, resulting in a decrease in the thermal stability of the subcrystalline region and double helix structure of starch in Indica rice flour. However, when the addition amount reaches 4%, the restrictive effect of KGM on water availability is enhanced, making the subcrystalline region and double helix structure of starch more difficult to degrade. A previous study has found that KGM addition amounts less than or equal to 3% had a similar reducing effect on the ΔH of rice starch [24].

### 3.2. Effect of Adding KGM on the Pasting Properties of Indica Rice Flour

**Figure 1:**
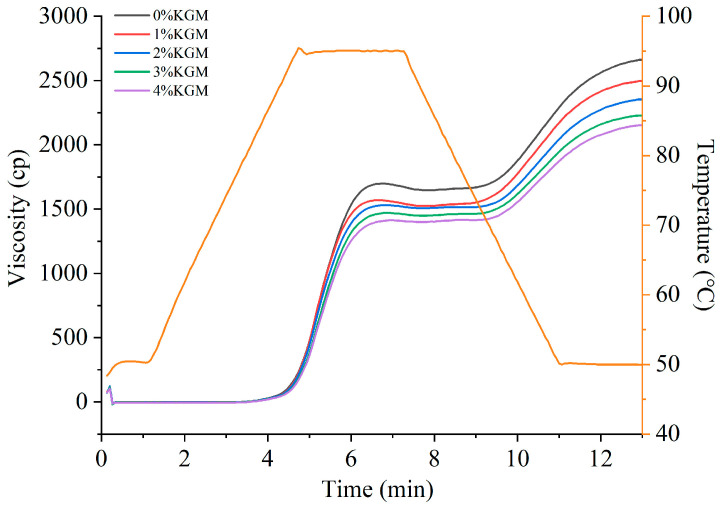
Effect of adding konjac glucomannan on the pasting curve of Indica rice flour.

**Figure 2:**
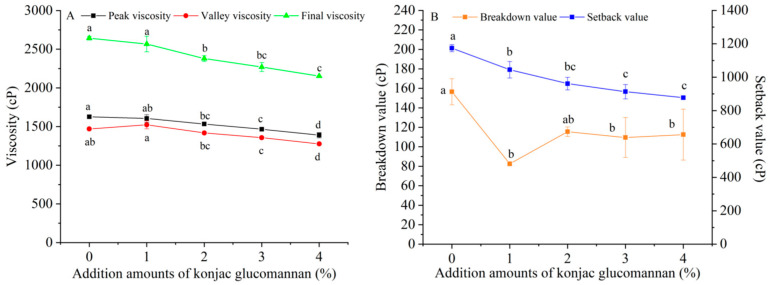
Effect of adding konjac glucomannan on the peak viscosity, valley viscosity, final viscosity (**A**), breakdown value, and setback value (**B**) of Indica rice flour. Different letters on data points of the same color indicate significant differences (*p* < 0.05).

The pasting properties of Indica rice flour were analyzed using an RVA. As shown in Figure 1, the pasting curve of Indica rice flour moved downward with the increase in KGM addition amount, but the pasting temperature basically remained at about 92 °C. The peak viscosity, valley viscosity, final viscosity, and setback value of Indica rice flour in the process of pasting reached the lowest value with the increase in KGM addition amount to 4%, which decreased by 14.6%, 13.2%, 18.6%, and 25.3%, respectively (Figure 2). In addition, the breakdown value also decreased when adding KGM, but the decrease degree did not significantly change with the increase in KGM addition amount.

In the RVA test, the weight ratio of Indica rice flour to water was 1:10, and the heating temperature was up to 95 °C, which provided sufficient water and heat for starch gelatinization, so that the system viscosity changed regularly with the absorption, expansion and disintegration of starch granules. On this condition, with sufficient water, KGM can dissolve in water to form a continuous phase that wraps starch granules inside, thereby acting as a physical protector to reduce the frictional degradation of starch granules, thus reducing the viscosity and breakdown value of Indica rice flour during the pasting process of the starch in it. Previous studies have found that KGM has a similar effect on the pasting properties of extruded yam starch [25] and low-amylose rice starch [22].

In addition, during the pasting process of the starch in Indica rice flour, KGM, which exists as a continuous phase, obviously has an isolating effect on the dispersed starch molecules. Furthermore, with sufficient water, starch molecules separated by KGM are more likely to undergo hydrogen bonding interaction with KGM [22] and are less likely to aggregate to form retrogradation structures. Therefore, the setback value that used to reflect the short-term retrogradation of the gelatinized starch during cooling will decrease due to the addition of KGM. A previous study has shown that adding KGM can inhibit the short-term retrogradation of amylose [26].

### 3.3. Effect of Adding KGM on the Rheological Properties of Indica Rice Flour Paste

**Figure 3:**
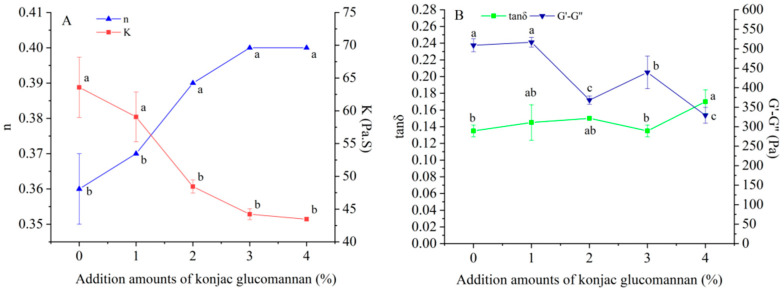
Effect of adding konjac glucomannan on the flow properties (**A**) and viscoelasticity (**B**) of Indica rice flour paste. Different letters on data points of the same color indicate significant differences (*p* < 0.05).

The effect of adding KGM on the rheological properties of Indica rice flour paste was analyzed using the paste samples formed in the RVA test. The flow properties of the samples were described using the flow behavior index n and the consistency coefficient K of the Herschel–Bulkley model with fit index R^2^ ranging from 0.9978 to 0.9990. As shown in Figure 3A, all samples had n values less than 1, and when the KGM addition amount exceeded 1%, the n value significantly increased while the corresponding K value significantly decreased. The results indicate that the KGM addition amount of more than 1% significantly reduced pseudoplasticity and viscosity of the Indica rice flour paste. The weakening of pseudoplasticity implys that the stability of the paste was improved, and the improvement is likely due to the hydrogen bonding interaction between KGM and starch molecules increasing with the KGM addition amount. In addition, the decreasing trend in adding KGM to the paste viscosity was consistent with that of the final viscosity of Indica rice flour during pasting.

Viscoelasticity of Indica rice flour paste was described using loss tangent (tan δ) and difference between storage modulus G′ and loss modulus G″ (G′-G″). As shown in Figure 3B, all samples had tan δ values less than 1, and when the KGM addition amount exceeded 1%, the value of G′-G″ significantly decreased. The results indicate that all the paste samples exhibited elasticity-dominated gel rheology characteristics, and the elasticity of Indica rice flour paste was significantly reduced by KGM addition amount of more than 1%. The decreasing trend in adding KGM to the paste elasticity was consistent with that of the setback value of Indica rice flour during pasting, supporting the inhibitory effect of KGM on the short-term retrogradation of the gelatinized starch.

### 3.4. Effect of Adding KGM on the Structure of Instant Dry Rice Noodles Made of Indica Rice Flour

**Figure 4:**
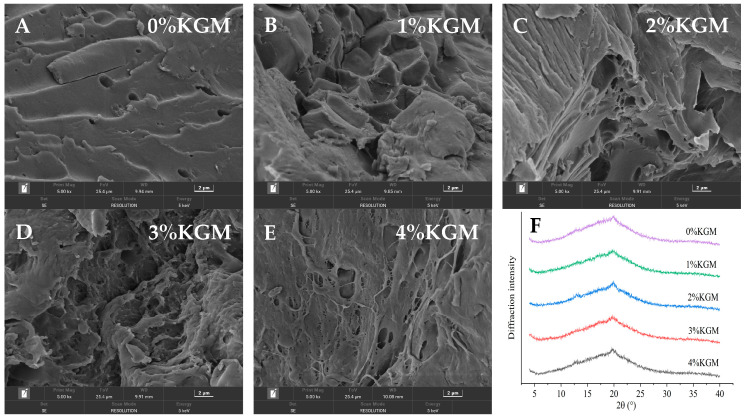
Effect of adding konjac glucomannan on the SEM images (**A**–**E**) and XRD patterns (**F**) of instant dry rice noodles made of Indica rice flour.

On the basis of finding that the addition of KGM can significantly change in starch gelatinization characteristics, pasting properties, and rheological properties of Indica rice flour, the structure of instant dry rice noodles made of Indica rice flour was analyzed to see whether the addition of KGM can help to form a structure conducive to improving the rehydration characteristics of instant dry rice noodles. SEM images and XRD patterns of all instant dry rice noodles are shown in Figure 4. Under a SEM, instant dry rice noodles without KGM had a solid structure with a few holes (Figure 4A). By comparison, instant dry rice noodles with KGM had a looser structure with a large number of holes or flake structures (Figure 4B–E), and the sample with 2% KGM showed the thinnest and lightest loose structure (Figure 4C). Under XRD, all the samples showed a V-type starch structure with a strong peak at 19.9° and a weak peak at 13.0° [27], indicating that the addition of KGM did not change the crystalline structure of starch in instant dry rice noodles.

**Figure 5:**
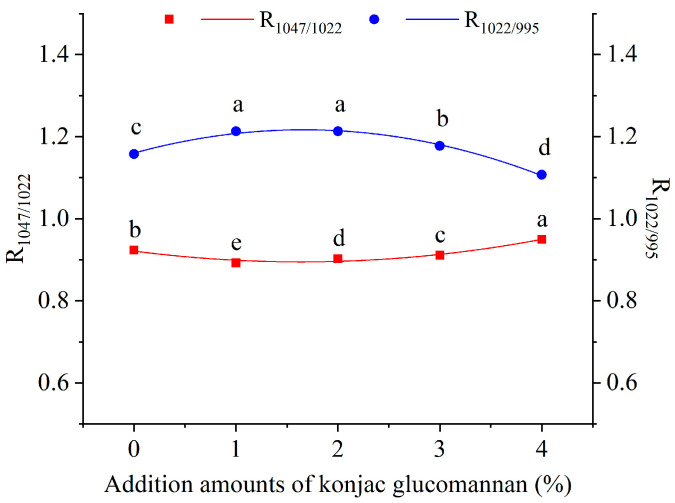
Effect of adding konjac glucomannan on the short-range ordered structure of instant dry rice noodles made of Indica rice flour. Different letters on data points of the same color indicate significant differences (*p* < 0.05).

In addition, FTIR spectroscopy analysis showed that the addition of KGM did not change the infrared peak positions of instant dry rice noodles (Figure S1) but significantly affected the peak intensity ratios of 1047 to 1022 (R_1047/1022_) and 1022 to 995 (R_1022/995_). As shown in Figure 5, with the increase in KGM addition amount, the R_1047/1022_ values of the samples showed a parabolic change with an opening upward (y = 0.00984x^2^ − 0.03221x + 0.092135, R^2^ = 0.95187), and the lowest R_1047/1022_ value corresponded to 1% KGM, while the R_1022/995_ values showed a parabolic change with an opening downward (y = −0.02049 + 0.0682x + 1.16011, R^2^ = 0.9935), and the highest R_1022/995_ value corresponded to 2% KGM. R_1047/1022_ can be used to evaluate the short-range orderliness of starch structure, with a higher value indicating better short-range orderliness, while R_1022/995_ can be used to estimate the double helix content of starch, with a higher value indicating lower double helix content [28]. Therefore, adding 1~3% KGM can significantly reduce the short-range ordered structure and double helix content of starch in instant dry rice noodles. The results support the DSC finding that adding less than or equal to 3% KGM can reduce the thermal stability and double helix structure of starch in Indica rice flour and are consistent with the RVA and rheological research findings that adding KGM can inhibit the short-term retrogradation of gelatinized starch in Indica rice paste.

According to the above effect of adding KGM on the structure of instant dry rice noodles, adding 2% KGM is most likely to make instant dry rice noodles have better rehydration properties.

### 3.5. Effect of Adding KGM on the Food Quality of Instant Dry Rice Noodles Made of Indica Rice Flour

The rehydration properties, texture characteristics, and sensory quality of all the noodles were analyzed to see the effect of adding KGM on the food quality of instant dry rice noodles made of Indica rice flour.

**Figure 6:**
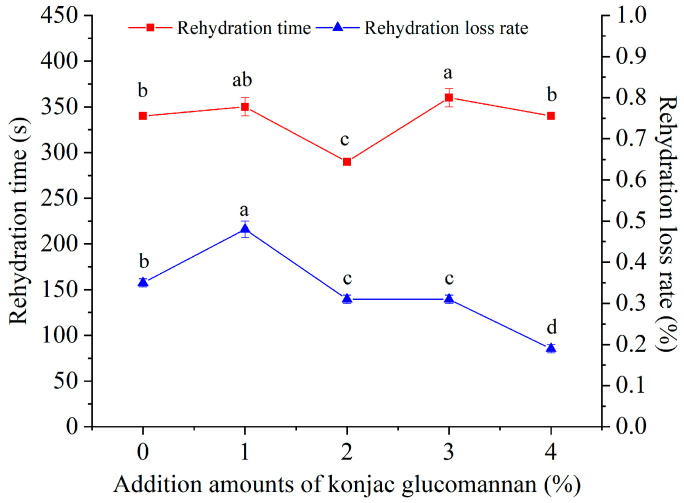
Effect of adding konjac glucomannan on the rehydration time and rehydration loss rate of instant dry rice noodles made of Indica rice flour. Different letters on data points of the same color indicate significant differences (*p* < 0.05).

As shown in Figure 6, KGM addition amounts of more than 1% and less than 4% significantly changed the rehydration time of instant dry rice noodles, and adding 2% KGM shortened the rehydration time from 340 s to the minimum of 290 s, with a reduction rate of 14.7%. In addition, during the rehydration process, the rehydration loss rate of instant dry rice noodles increased first and then decreased with the increase in the KGM addition amount. The rehydration loss rate was highest at 0.48% when the KGM addition amount was 1% and lowest at 0.19% when the KGM addition amount was 4% with a variation of only 0.29%. Specifically, when the KGM addition amount was 2%, the rehydration loss rate of instant dry rice noodles was reduced from 0.35% to 0.31%, and the reduction rate was 11.4%. Therefore, considering both the rehydration time and rehydration loss rate, adding 2% KGM can most effectively improve the rehydration properties of instant dry rice noodles. According to the effect of adding KGM on the structure of instant dry rice noodles, the reason for this result is that instant dry rice noodles with 2% KGM had the thinnest and lightest loose structure and the starch structure with low short-range order and double helix content.

**Figure 7:**
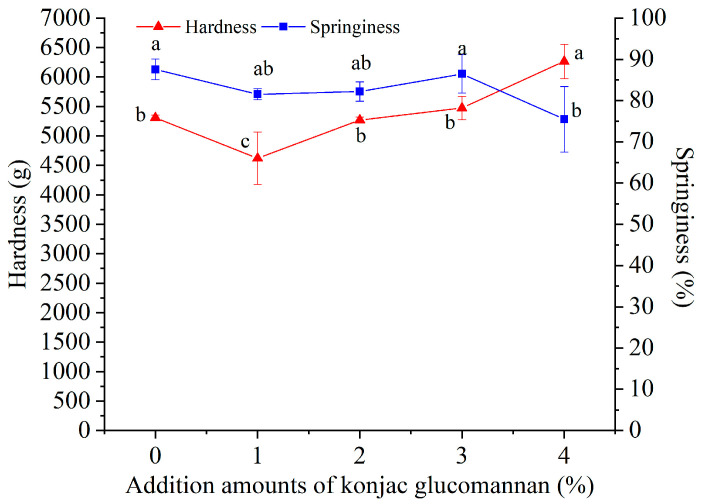
Effect of adding konjac glucomannan on the texture characteristics of instant dry rice noodles made of Indica rice flour. Different letters on data points of the same color indicate significant differences (*p* < 0.05).

For the texture characteristics, there was no significant effect of adding KGM on the resilience, adhesiveness, cohesion, and chewiness of instant dry rice noodles after rehydration, but the hardness and springiness were changed by adding KGM. As shown in Figure 7, the hardness was decreased by adding 1% KGM and increased by adding 4% KGM, while the springiness was decreased by adding 4% KGM. However, adding 2% KGM did not significantly change the hardness and springiness of instant dry rice noodles after rehydration.

**Figure 8:**
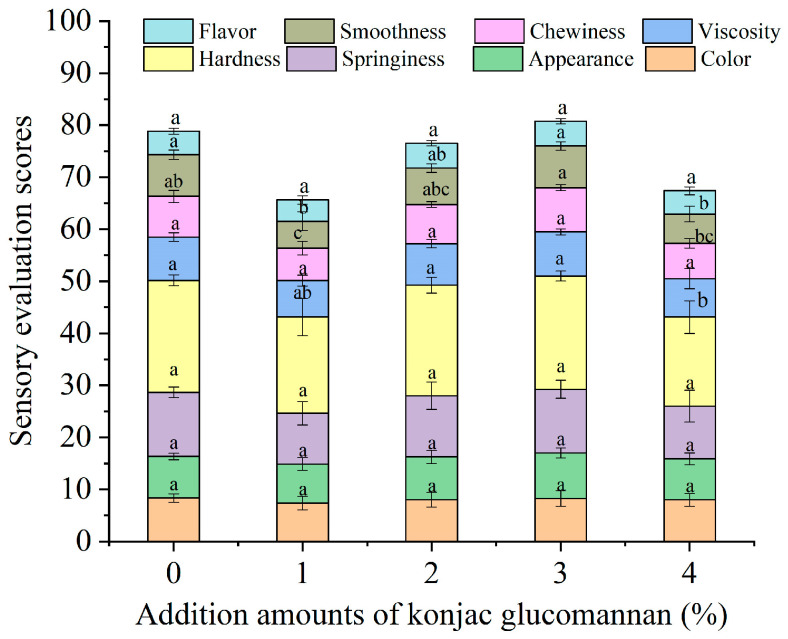
Effect of adding konjac glucomannan on the sensory properties of instant dry rice noodles made of Indica rice flour. Different letters within each color bar indicate significant differences (*p* < 0.05).

For the sensory quality, as shown in Figure 8, the total sensory evaluation score of instant dry rice noodles after rehydration was significantly reduced by adding 1% and 4% KGM. Specifically, the chewiness was reduced by adding 1% KGM, the hardness was reduced by adding 4% KGM, and the smoothness was reduced by adding 1% and 4% KGM. However, adding 2% and 3% KGM did not significantly change the sensory quality of instant dry rice noodles.

Obviously, the effect of adding KGM on the food quality of instant dry rice noodles made of Indica rice flour is as complex as that on the physicochemical properties of Indica rice flour and the structure of instant dry rice noodles. However, in accordance with these complex effects, adding 2% KGM can improve the rehydration properties of instant dry rice noodles without affecting their texture and sensory quality. A previous study has shown that adding an appropriate amount of KGM can improve the quality of surimi-wheat dough and noodles [29].

### 3.6. Effect of Adding KGM on the Starch Digestibility of Instant Dry Rice Noodles Made of Indica Rice Flour

**Figure 9:**
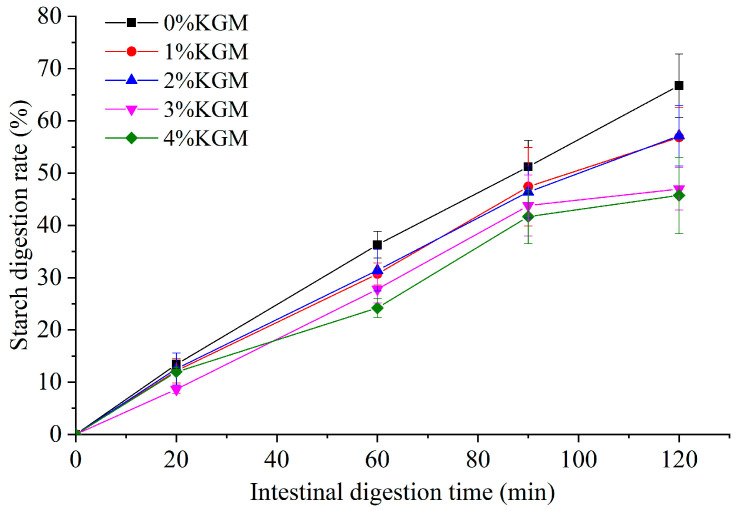
Effect of adding konjac glucomannan on the starch digestion rate of instant dry rice noodles made of Indica rice flour.

**Figure 10:**
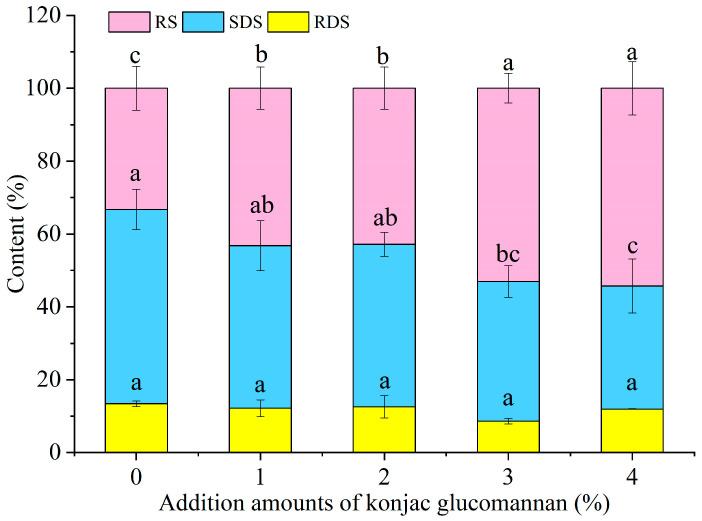
Effect of adding konjac glucomannan on the starch digestion type of instant dry rice noodles made of Indica rice flour. Different letters within each color bar indicate significant differences (*p* < 0.05).

The digestion rate and type of starch in all the noodles after rehydration were determined using the in vitro digestion method to see the effect of adding KGM on the starch digestibility of instant dry rice noodles made of Indica rice flour. As shown in Figure 9, in general, the starch digestion rate of instant dry rice noodles decreased with the increase in the KGM addition amount, but there was no significant difference in the effect of adding 2% and 3% KGM. In addition, as shown in Figure 10, the RDS content of instant dry rice noodles did not significantly change due to the addition of KGM, but overall, the SDS content decreased with the increase in the KGM addition amount, while the RS content was the opposite. Specifically, when 2% KGM was added, the SDS content of instant dry rice noodles decreased from 53.33% to 44.61% with a decrease rate of 16.4%, while the RS content increased from 33.26% to 42.85% with an increase rate of 28.8%. Previous studies have found that KGM has similar effects on the SDS and RS contents of rice starch [24] and extruded yam starch [25].

The inhibitory effect of adding KGM on the starch digestibility of instant dry rice noodles made of Indica rice flour was consistent with the finding that KGM inhibited the enzymatic hydrolysis of starch reported by Sasaki et al. [5]. According to the processing technology of the instant dried rice noodles, during the steaming and extruding process after mixing with water, the KGM dissolved in water will wrap the gelatinized Indica rice flour, and the wrapping compactness will increase with the increase in the KGM content. Furthermore, the wrapping effect will be strengthened by the subsequent drying process, making it more difficult for starch molecules to be hydrolyzed by amylase.

## 4. Conclusions

Adding KGM can inhibit the short-term retrogradation of the gelatinized starch in Indica rice flour and result in a decrease in starch digestibility and an increase in resistant starch content of its instant dry rice noodles. Adding 2% KGM can improve the rehydration properties of instant dry rice noodles without affecting their texture or sensory quality. The research findings can promote the application of KGM in improving the quality of rice products.

## Figures and Tables

**Table 1:** Sensory evaluation criteria for instant dry rice noodles.

Evaluating Indicators	Evaluation Criteria
Color (10 points)	It refers to the color and brightness of rice noodles. Light white, glossy, without obvious spots, shiny (8–10 points); average brightness (7–4 points); dark, gray, poor brightness (1–3 points).
Appearance (10 points)	It refers to the smoothness and expansion of rice noodles. Uniform thickness, delicate tissue, smooth surface (8–10 points); smooth surface with a small amount of impurities and slight cracks (5–7 points); rough surface with many impurities and cracks (2–4 points).
Hardness (25 points)	It refers to the force required to bite rice noodles with teeth. Suitable for softness and hardness (21–25 points); slightly soft or slightly hard (14–20 points); very soft or very hard (0–13 points).
Springiness (20 points)	It refers to the strength and springiness of rice noodles when chewing. Good springiness (15–20 points); moderate springiness (8–14 points); poor springiness (3–7 points).
Viscosity (10 points)	It refers to the degree of rice noodles sticking to teeth during chewing. Refreshing and not sticking to teeth (8–10 points); slightly sticking to teeth (5–7 points); sticking to teeth (2–4 points).
Smoothness (10 points)	It refers to the smoothness of the taste of rice noodles. Smooth and refreshing (8–10 points); relatively smooth (4–7 points); rough and not refreshing (0–3 points).
Chewiness (10 points)	It refers to the chewing resistance of rice noodles. Durable to chew, good taste (8–10 points); relatively chewable, average taste (5–7 points); not chewable, poor taste (2–4 points).
Flavor (5 points)	It refers to the flavor of rice noodles. Having fragrant aroma of rice (5 points); basically, no peculiar smell (3–4 points); having peculiar smell (1–2 points).

**Table 2:** Effect of adding konjac glucomannan on the starch gelatinization parameters of Indica rice flour.

Addition Amounts of Konjac Glucomannan (%)	T_o_ (°C)	T_p_ (°C)	T_c_ (°C)	ΔH (J/g)
0	70.51 ± 0.52 ^a^	76.25 ± 0.06 ^a^	82.51 ± 0.45 ^a^	3.25 ± 0.11 ^b^
1	70.17 ± 0.19 ^a^	75.45 ± 0.88 ^a^	81.57 ± 0.19 ^ab^	2.53 ± 0.48 ^c^
2	69.75 ± 2.06 ^a^	74.95 ± 0.23 ^a^	81.02 ± 0.32 ^b^	2.74 ± 0.08 ^bc^
3	70.30 ± 2.32 ^a^	75.34 ± 0.95 ^a^	80.73 ± 0.86 ^b^	2.37 ± 0.18 ^c^
4	68.48 ± 0.39 ^a^	75.11 ± 0.41 ^a^	82.66 ± 0.33 ^a^	4.45 ± 0.23 ^a^

Different letters in the same column indicate significant differences (*p* < 0.05).

## Data Availability

The original contributions presented in this study are included in the article/Supplementary Materials; further inquiries can be directed to the corresponding authors.

## Supplementary Materials

The following supporting information can be downloaded at: https://www.mdpi.com/article/10.3390/foods13233749/s1, Figure S1. Effect of adding konjac glucomannan on FTIR pattern of instant dry rice noodles made of the indica rice flour.
