# Supplementary material for: Effect of Adding Konjac Glucomannan on the Physicochemical Properties of Indica Rice Flour and the Quality of Its Product of Instant Dry Rice Noodles
Source: Foods. 2024 Nov 22;13(23):3749. doi: 10.3390/foods13233749 (PMC11640252; doi:10.3390/foods13233749)
Supplement: Supplementary file 1 [file foods-13-03749-s001.zip › foods-3307078-supplementary.pdf]

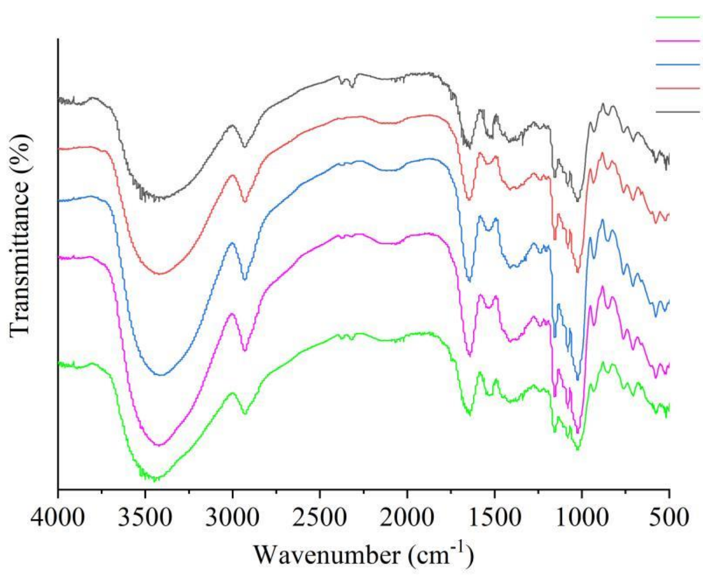

Figure S1. Effect of adding konjac glucomannan on FTIR pattern of instant dry rice noodles made of the indica rice flour.
